# EWS/ETS Regulates the Expression of the Dickkopf Family in Ewing Family Tumor Cells

**DOI:** 10.1371/journal.pone.0004634

**Published:** 2009-02-27

**Authors:** Yoshitaka Miyagawa, Hajime Okita, Mitsuko Itagaki, Masashi Toyoda, Yohko U. Katagiri, Junichiro Fujimoto, Jun-ichi Hata, Akihiro Umezawa, Nobutaka Kiyokawa

**Affiliations:** 1 Department of Developmental Biology, National Research Institute for Child Health and Development, Setagaya-ku, Tokyo, Japan; 2 Vice President General, National Research Institute for Child Health and Development, Setagaya-ku, Tokyo, Japan; 3 Department of Reproductive Biology, National Research Institute for Child Health and Development, Setagaya-ku, Tokyo, Japan; Northwestern University, United States of America

## Abstract

**Background:**

The Dickkopf (DKK) family comprises a set of proteins that function as regulators of Wnt/β–catenin signaling and has a crucial role in development. Recent studies have revealed the involvement of this family in tumorigenesis, however their role in tumorigenesis is still remained unclear.

**Methodology/Principal Findings:**

We found increased expression of DKK2 but decreased expression of DKK1 in Ewing family tumor (EFT) cells. We showed that EFT-specific EWS/ETS fusion proteins enhance the DKK2 promoter activity, but not DKK1 promoter activity, via ets binding sites (EBSs) in the 5′ upstream region. EWS/ETS-mediated transactivation of the promoter was suppressed by the deletion and mutation of EBSs located upstream of the DKK2 gene. Interestingly, the inducible expression of EWS/ETS resulted in the strong induction of DKK2 expression and inhibition of DKK1 expression in human primary mesenchymal progenitor cells that are thought to be a candidate of cell origin of EFT. In addition, using an EFT cell line SK-ES1 cells, we also demonstrated that the expression of DKK1 and DKK2 is mutually exclusive, and the ectopic expression of DKK1, but not DKK2, resulted in the suppression of tumor growth in immuno-deficient mice.

**Conclusions/Significance:**

Our results suggested that DKK2 could not functionally substitute for DKK1 tumor-suppressive effect in EFT. Given the mutually exclusive expression of DKK1 and DKK2, EWS/ETS regulates the transcription of the DKK family, and the EWS/ETS-mediated DKK2 up-regulation could affect the tumorigenicity of EFT in an indirect manner.

## Introduction

The Wnt/β–catenin signaling pathway is known to regulate development, differentiation, and a variety of biological phenomena. Recent findings support notion that the aberration of canonical Wnt/β–catenin signaling is involved in malignant transformation [1], [2], [3]. Mutations in components of the pathway have been observed in primary human cancers. These mutations often allow ligand-independent Wnt/β–catenin signaling in tumor cells. Among the components, the tumor suppressor Adenomatous polyposis coli (APC) and the scaffold protein Axin are frequently mutated in colon cancer [4] and hepatocellular carcinoma [5] respectively. Mutations in β–catenin itself are also found in a number of cancers. These changes induce the stabilization of β–catenin in the cytoplasm and an abnormal accumulation of free β–catenin in the nucleus, resulting in the aberrant activation of Wnt target genes through T-cell factor family members.

A number of activators and antagonists in the Wnt/β–catenin signaling pathway have been cloned and investigated. The Dickkopf (DKK) family is comprised of secreted protein modulators of Wnt/β–catenin signaling [6], [7]. In human, the family consists of DKK1, DKK2, DKK3/REIC and DKK4, all of which have two cysteine-rich domains. DKK1 interacts with low-density lipoprotein receptor (LRP) 5/6, a component of the Wnt receptor complex, and inhibits canonical Wnt/β–catenin signaling (Mao et al. 2001). DKK2 is structurally very similar to DKK1 and also interacts with LRP5/6, but its effect on Wnt/β–catenin signaling is thought to be rather agonistic [8], [9]. DKKs have been found to be important in multiple developmental processes such as limb development [10], [11], [12] and bone formation [13], [14].

In addition, it has been recently reported that DKKs play a crucial role in cell transformation [15]. Hyper-methylation of the promoter and gene silencing of DKK1 were observed in tumor cells, including colorectal cancer [16] and malignant melanoma cells [17]. Given evidence that ectopic expression of DKK1 suppresses features of transformation in tumor cells [18], [19], [20], DKK1 might inhibit tumorigenicity. However, the expression of DKK1 is elevated in some tumor cells including myeloma cells [21], hepatoblastoma cells and Wilm's tumor cells [22]. Therefore, the molecular function of DKK1 in cancer is controversial and still not fully elucidated. DKK3/REIC is also proposed as a tumor suppressor. The overexpression of DKK3/REIC inhibits tumor growth in prostate cancer [23], melanoma [17] and hepatocellular carcinoma [24]. The down-regulated expression of DKK3/REIC in osteosarcoma [25], [26], hepatoblastma [26] and prostate cancer [27] further supports this notion. Although these studies indicate that the modulation of DKK expression contributes to tumorigenicity, the underlying molecular mechanism is not fully understood.

Ewing family tumor (EFT) is a pediatric cancer arising from bone and soft tissues. In EFT, a specific translocation results in production of the fusion protein EWS/ETS, where the C-terminal of EWS, including the RNA-binding domain, is replaced with a DNA-binding domain of the ets gene family, such as FLI1, ERG, E1AF, ETV1 and FEV [28]. The consequent fusion proteins have been proposed to act as an aberrant transcriptional regulator and believed to play an important role in the initiation and development of EFT. EWS/FLI1, transactivates the expression of cyclin D1 [29], cyclin E [30] and TERT [31] through the Sp1, E2F or ets DNA-binding sites located in each promoter, but suppresses the expression of p21 [32] and TGFBRII [33], [34].

In this paper, we present evidence of enhanced DKK2 but suppressed expression of DKK1 in EFT cells. The experiments including those using inducible EWS/ETS expression systems in human primary bone marrow-derived mesenchymal progenitor cells (hMPCs) [35] demonstrated that the expression of DKKs is regulated by the EFT-specific chimeric protein, EWS/ETS. We further address the role of DKKs in the tumorigenicity of EFT.

## Materials and Methods

### Animals

All the animals used in this study were treated in accordance with regulation on Animal Experimentation at National Research Institute for Child Health and Development.

### Plasmid construction

To construct a luciferase reporter vector using the 5′upstream region of the DKK2 gene, the −1955/+49 genomic fragment of the gene was amplified by PCR from human lymphocyte genomic DNA and cloned into the *EcoR*V site of the reporter vector pGL4 (Promega) to generate pGL4-DKK2. Serial deletions of pGL4-DKK2 were generated by digestion with restriction enzymes and subsequent self-ligation. The resultant reporter vectors were designated pGL4-DKK2Δ*Kpn*I (−1741/+49), pGL4-DKK2Δ*Nhe*I (−1241/+49) and pGL4-DKK2Δ*Sac*I (−521/+49). Mutagenesis of putative ets binding sites (EBS) in the DKK2 5′upstream region was performed using KOD-plus (TOYOBO). The primers used for the mutagenesis were as follows: for the −1585/−1573 genomic fragment of the DKK2 5′upstream region (designated EBS-1): 5′-CTACCTTAAA GAAACCTTAT TCAAAAGATA3′ and 5′-AGATTTTTCA CATTTTAGTG TGTGGGGTTT-3′; for the −904/−895 genomic fragment of the DKK2 5′upstream region (designated EBS-2): 5′-GCACCTTGCC AAGGAAGACA GGATCTCAAA-3′ and 5′-CTTCTAGCCC CAGTGAATTA CAAGAGAAGC-3′. A flag-tag and a Gateway cassette were amplified from pifw [36] by PCR and the product was inserted into the *EcoR*V site of pcDNA™3 (Invitrogen) (termed pcDNA3-flagDEST). Full-length EWS/FLI1 type II, EWS/ERG and EWS/E1AF cDNAs were amplified from cDNAs prepared from NCR-EW2 [37], W-ES [38] and NCR-EW3 cells [37], respectively, by PCR and cloned into the *Xmn*I-*EcoR*V sites of pENTR™11 (Invitrogen). The resulting pENTR11-EWS/ETSs were recombined with pcDNA3-flagDEST using the LR recombination reaction as instructed by the manufacturer (Invitrogen) to construct the flag-tagged EWS/ETS expression vector pcDNA3-flagEWS/ETSs. The human DKK1 and DKK2 cDNAs without a stopcodon were subcloned into pENTR™11 and the resulting pENTR11-DKK1 and pENTR11-DKK2 were recombined with pcDNA-DEST40 C-terminally V5-His-tagged (Invitrogen) using the LR recombination reaction as instructed by the manufacturer, to obtain pcDNA-DEST40-DKK1 and -DKK2.

### Cell cultures

H4-1 (primary hMPCs), UEET-12, UET-13 (hMPCs with an extended life span through retroviral transduction) [39] and UET-13 transfectants [35] were cultured in Dulbecco's modified Eagle's medium (DMEM) with 10% Fetal Bovine Serum (FBS) or Tet System Approved FBS (Takara) at 37°C under a humidified 5% CO_2_ atmosphere. The EFT cell lines EES-1, SCCH196, RD-ES, SK-ES1, NCR-EW2, NCR-EW3 and W-ES, neuroblastoma (NB) cell lines NB9, NB69 and GOTO, and Rhabdomyosarcoma (RMS) cell line NRS-1 were maintained as described previously [35]. Other NB cell lines NB1, NB19 [40], CHP134 and IMR32 [41], the Burkitt lymphoma (BL) cell lines BJAB, Ramos, Daudi, P3HR1 and Raji (obtained from Japanese Collection of Research Bioresources, JCRB, Osaka, Japan) were cultured in RPMI 1640 medium with 10% FBS. Embryonal carcinoma (EC) cell lines NCR-G2 and NCR-G3 [42] were cultured in a 1∶1 mixture of DMEM and Ham's F12 medium with 10% FBS.

HEK293 cells were cultured in DMEM with 10% FBS. Stable transfection of HEK293 cells with pcDNA3-flagDEST or pcDNA3-flagEWS/FLI1 was performed using Lipofectamine™ 2000 (Invitrogen) according to the manufacturer's directions. Individual resistant clones were selected in the presence of 1000 µg/ml of G418 for a month and designated as HEK-DEST and HEK-EWS/FLI1 cells.

For the generation of SK-ES1 transfectants with DKK1 and DKK2, SK-ES1 cells transfected with pcDNA-DEST40-DKK1 and -DKK2, respectively and selected as described above. The consequent stable transfectants were designated SK-ES1-DKK1 and -DKK2, respectively. As a negative control, SK-ES1 cells were also transfected with empty vector pcDNA-DEST40 and designated as SK-ES1-DEST.

### Western blot analysis

The Western blot analysis was performed as described [43]. Briefly, cell lysate was prepared, separated on a 10% SDS-PAGE gel, and transferred onto a PVDF membrane. After blocking with 5% skim milk in PBS containing 0.01% Tween-20 (Sigma) (PBST), the membrane was incubated with appropriate primary and secondary antibodies. As the primary antibody, anti-flag M2 (Sigma), anti-V5 (Invitrogen) or anti-Actin (Sigma) was used. HRP-conjugated anti-rabbit or anti-mouse IgG antibody (DakoCytomation) was used as the secondary antibody. Blots were detected using an ECL Plus Western Blotting Detection System (GE Healthcare Bio-Science Corp) and exposed to X-ray film (Kodak) for 5–30 minutes. Secreted DKK2 was detected using anti-DKK2 antibody (Biovision). For the detection of secreted DKKs-V5 proteins, 20 ml of each cell culture supernatant in SK-ES1 transfectants was collected and added to BD TALON Metal Affinity Resin (Clontech). After incubation overnight, the supernatant was removed. The resin-protein complex was purified and analyzed by Western blotting as described above.

### Immunoprecipitation (IP)

IP was performed as described previously [44]. Whole cell extracts were incubated with anti-flag M2 antibody (Sigma) and the protein-antibody complex was bound to protein G-Dynal beads (Invitrogen). The bound complexes were washed and collected by centrifugation and separated by SDS-PAGE.

### Reporter assay

HEK293 cells were transiently transfected with a mixture of 100 ng of pGL4 or the pGL4-DKK2 reporter vector series and 300 ng of pcDNA3-flagDEST or pcDNA3-flagEWS/ETS using Lipofectamine™ 2000 (Invitrogen). The cells were also transfected with 25 ng of pcDNA/GW-40/LacZ (Invitrogen) for the standardization of transfection efficiency. The luciferase activity and β-gal activity were measured 48 h after the transfection as described before (Mon *et al.*, 2003).

### RT-PCR analysis

RT-PCR analysis was performed as described [35]. Total RNA was extracted from cells using a RNeasy kit (QIAGEN) and reverse transcribed using a First-Strand cDNA Synthesis Kit (GE Healthcare Bio-Science Corp). The primers used for the detection of DKK1 and DKK2 were as follows: DKK1 primers, 5′-CGCTAGTCCC ACCCGCGGAG GGGACGCAGG -3′ and 5′-CCTTCTTTCA GGACAGGTTT ACAGATCTTG -3′; DKK2 primers, 5′-GAAGCGCTGC CACCGAGATG GCATGTGCTG -3′ and 5′-GATGATCGTA GGCAGGGGTC TCCTTCATGC -3′). For the detection of DKK-V5 fusion transcripts, the following V5-specific primer was used: V5 (reverse): 5′-ACGCGTAGAATCGAGACCGAGAGAGGGTT-3′. The human GAPDH gene was detected as an internal control with the following primers: GAPDH (forward): 5′-CCACCCATGG CAAATTCCAT GGCA-3′, GAPDH (reverse): 5′-TCTAGACGGC AGGTCAGGT CCACC-3′. PCR products were electrophoresed with a 1% agarose gel and stained by ethidium bromide.

### Real-time RT-PCR analysis

Real-time RT-PCR was performed using SYBR® Green PCR Master Mix, TaqMan® Universal PCR Master Mix and TaqMan® Gene Expression Assays, Inventoried assay on an ABI PRISM® 7900HT Sequence Detection System (Applied Biosystems) according to the manufacturer's instructions. The human GAPDH gene was used as an internal control for normalization.

### Subcutaneous tumorigenicity assay

Immuno-deficient mice (CB17-SCID 8 weeks old, Clea Japan, Inc) were maintained under pathogen-free conditions. SK-ES1 transfectants were suspended in 100 µl of PBS and injected s.c. into mice. Cells were injected at a density of 1×10^7^ cells per body. Mice were monitored weekly and tumor diameter was measured with precision calipers. Mice were sacrificed after 28 days of monitoring.

### Chromatin immunoprecipitation (ChIP) assay

For preparing the antibody- protein G-Dynal beads (Invitrogen) complex, protein G-Dynal beads were washed with 0.5% BSA in PBS, and anti-flag antibody, anti-FLI1 antibody (Santa cruz) or mouse IgG was added. The beads mixtures were incubated and rotated overnight at 4°C. HEK-EWS/FLI1 cells or SK-ES1 cells were cross-linked by 1% formaldehyde for 10 minutes at room temperature. The medium was aspirated and the cells were lysed using cell lysis buffer A [50 mM HEPES pH 7.5, 140 mM NaCl, 1 mM EDTA, 10% glycerol, 0.5% NP-40, 0.25% Triton X-100, and Complete protease inhibitor cocktail (Roche)]. The cells were pelleted and resuspended in cell lysis buffer B [10 mM Tris-HCl pH 8.0, 200 mM NaCl, 1 mM EDTA, 0.5 mM EGTA, and Complete protease inhibitor cocktail]. Nuclei were then pelleted and lysed with cell lysis buffer C [10 mM Tris-Hcl pH 8.0, 100 mM NaCl, 1 mM EDTA, 0.5 mM EGTA, 0.1% deoxycholate, 0.5% N-lauroylsarcosine, Complete protease inhibitor cocktail] on ice. The nuclear lysates were sonicated and then precleared with salmon sperm DNA/ protein G-Dynal beads for 3 hours. The cleared supernatant was incubated with anti-flag antibody, anti-FLI1 antibody or mouse IgG- protein G-Dynal beads complex. Sixteen hours after the incubation, the immune complexes were collected with centrifugation and washed 7 times using RIPA buffer [50 mM HEPES pH 7.5, 500 mM LiCl, 1 mM EDTA, 1% NP-40, 0.7% N-lauroylsarcosine, and Complete protease inhibitor cocktail]. The DNA-protein cross-linking was reversed by heating at 65°C. The chromatin was purified and analyzed by PCR using specific primers (EBS-2: 5′-GCAGGAAGCC AAGGAAGACA-3′ and 5′-GCGAATAGGA AATCCCAGAT AGG-3′). PCR products were electrophoresed with a 2% agarose gel and stained by SYBR® Green I (Takara).

## Results

### Increased DKK2 and suppressed DKK1 expression in EFT cells

To identify the specific genes related to EFT cells, DNA microarray-based global expression profiling was done using EFT cell lines and other tumors found in children. By comparing the expression patterns, genes were identified as typically up-regulated in EFT cells and other cells (data not shown). Among these genes, we particularly focused on DKK2 (up-regulated) and DKK1 (down-regulated) because they are members of the DKK family and structurally well related, but revealed opposite expression patterns in EFT cells. Similar results were obtained from the microarray assay using EFT and NB tissue samples in the Gene Expression Omnibus database GEO (Accession number: GSE1825) (data not shown).

**Figure 1:**
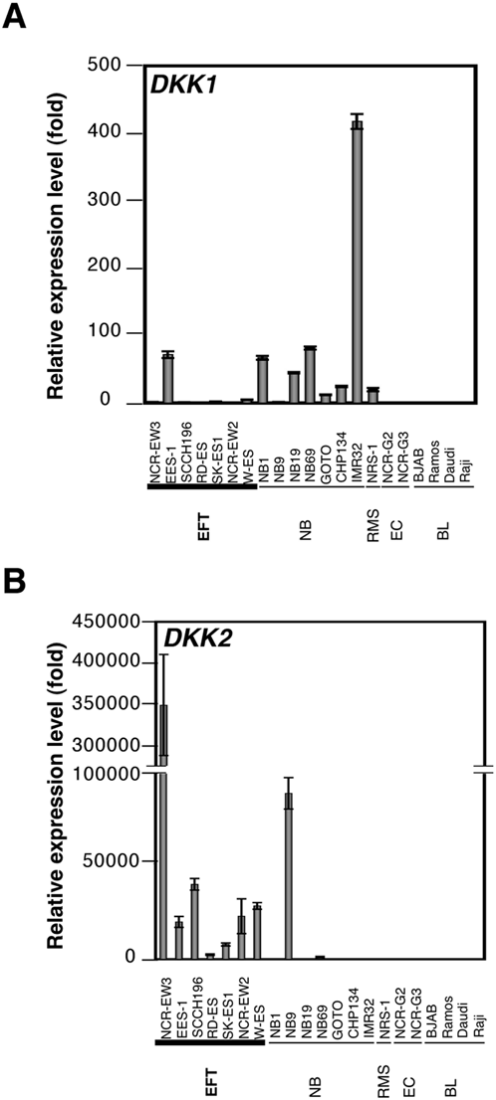
The expression pattern of the DKK family in Ewing's family tumor (EFT). A, B, Real-time RT-PCR analysis using pediatric tumor cell lines for DKK1 (A) and DKK2 (B) expression. EFT: Ewing's family tumor, NB: neuroblastoma, BL: Burkitt lymphoma, RMS: Rhabdomyosarcoma, EC: embryonal carcinoma. Data are normalized to the mRNA level in SCCH196 (for DKK1) and Ramos (for DKK2) which is arbitrarily set to 1. Signal intensity was normalized using that of a control housekeeping gene (human GAPDH gene). Data are relative values with the SD for triplicate wells.

We first confirmed the expression pattern of DKK genes obtained with the microarray assay by conducting a Real-time RT-PCR analysis. The increased expression of DKK2 was confirmed in EFT cells (Fig. 1A). DKK2 expression was especially increased in NCR-EW3 cells that express EWS/E1AF. In contrast, the expression of DKK1 was suppressed in most EFT cells (Fig. 1B).

### Direct regulation of DKK2, but not DKK1, promoter activity by EWS/ETS proteins

**Figure 2:**
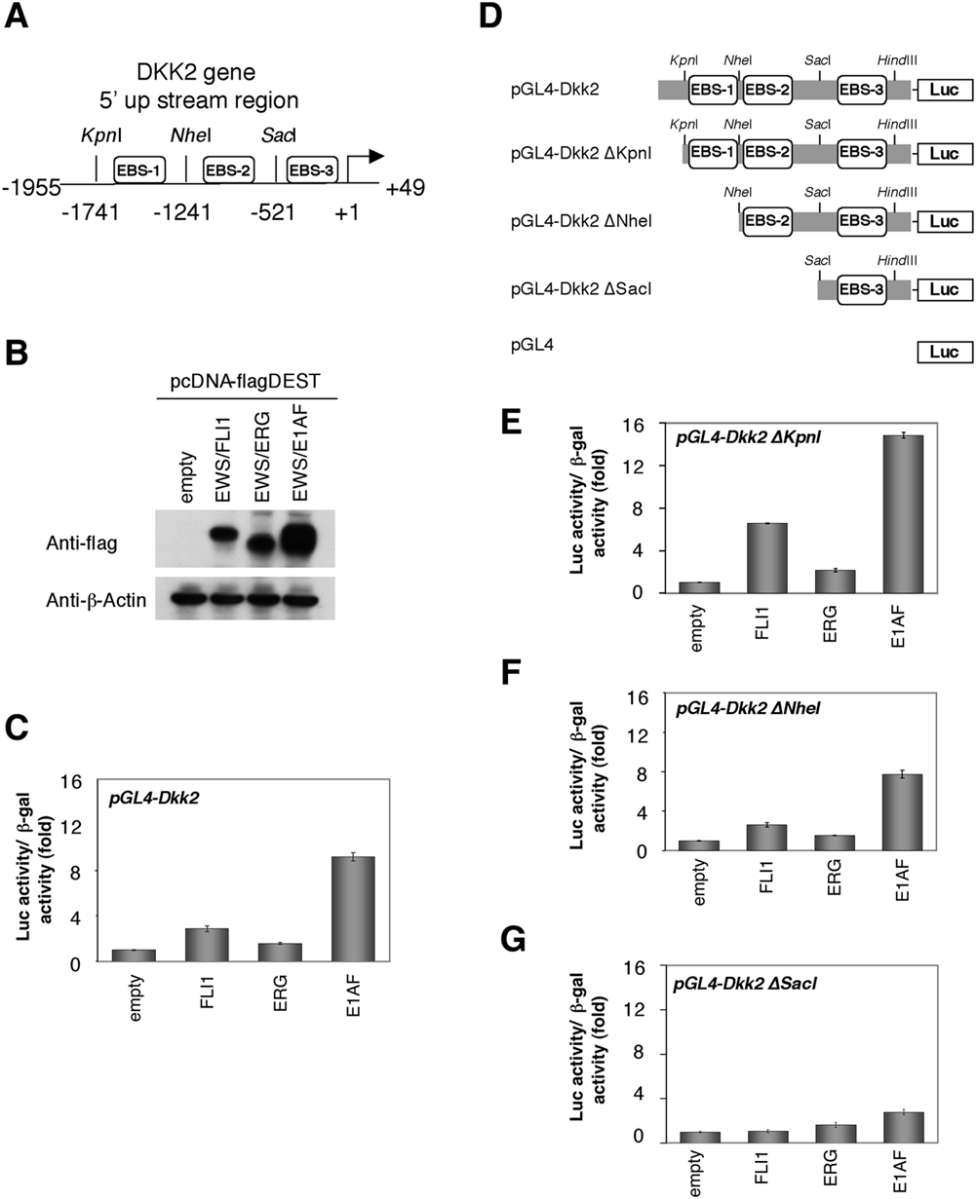
The effect of EWS/ETS expression on the activity of the DKK2 promoter. A, Schematic representation of the 5′upstream region of the DKK2 gene. The nucleotide numbering represents the distance from the translation start site (+1). *Kpn*I (−1741), *Nhe*I (−1241), and *Sac*I (−521) sites were used to construct a series of deletion mutants of the DKK2 promoter (see Materials and Methods). Consensus ets binding sites are boxed (EBS-1, EBS-2 and EBS-3). B, Western blot analysis for EWS/ETS expression. HEK293 cells were transiently transfected with pcDNA3-flagDEST or pcDNA3-flagEWS/ETS and then analyzed by anti-flag Western blotting. C, The effect of EWS/ETS expression on the activity of the DKK2 promoter. HEK293 cells were co-transfected with reporter plasmids and the expression vector. Luciferase activity was measured after 48 hours. Data are relative values with the SD for triplicate wells. Data are normalized to the value for the empty vector (pcDNA3-flagDEST) which is arbitrarily set to 1. The transfection efficiency was normalized relative to the β–galactosidase activity (See Materials and Methods). D, Schematic representation of a series of deletion mutants of the DKK2 5′ upstream region. KpnI (−1741), NheI (−1241) and SacI (−521) sites were used to construct a series of deletion mutants of the DKK2 promoter (see Materials and Methods). E, F, G, The transactivation of deletion mutants of the DKK2 promoter by EWS/ETS expression. HEK293 cells were co-transfected with reporter plasmids and the expression vector. Data are normalized to the value for the empty vector (pcDNA3-flagDEST) which is arbitrarily set to 1. Luciferase activity was measured after 48 hours. Data are relative values with the SD for triplicate wells. The transfection efficiency was normalized relative to the β–galactosidase activity (See Materials and Methods).

Previous reports revealed that EWS/ETS proteins enhance the promoter activity of several genes via the ets DNA-binding sites on the promoters [45]. A sequence analysis and TF search (http://www.cbrc.jp/research/db/TFSEARCH.html) revealed the 5′ upstream region (−1955 to +49) of the DKK2 gene to have two putative EBSs, here designated EBS-1 (−1585 to −1573) and EBS-2 (−904 to −895). Although, the TF search did not recognize it, we also found a putative EBS (GGAA/T) element, designated as EBS-3 (−140 to −131). Thus, we next isolated the 5′ upstream region of the DKK2 gene and cloned it into the reporter plasmid pGL4 (DKK2 full-Luc) (Fig. 2A) to test the effects of EWS/ETS expression on DKK2 promoter activity with transient transfection reporter assays in HEK293 cells. As shown in Fig. 2B and C, co-transfection of EWS/ETS with pGL4-DKK2 resulted in an enhancement of reporter activity ∼10-fold compared with co-transfection with empty expression vector. To map the site in the DKK2 5′ upstream region required for activation by EWS/ETS expression, reporter assays using a deletion series of pGL4-DKK2 were performed. Co-transfection of the EWS/ETS expression vector with DKK2 pGL4-DKK2Δ*Kpn*I or -DKK2Δ*Nhe*I resulted in a strong induction of the reporter activity (∼15-fold) (Fig. 2D–F), whereas that with pGL4-DKK2Δ*Sac*I led to only a faint enhancement (Fig. 2G), indicating that the sequence from -521 to +49 in the DKK2 gene is not sufficient for EWS/ETS-mediated enhancement of DKK2 transcription.

**Figure 3:**
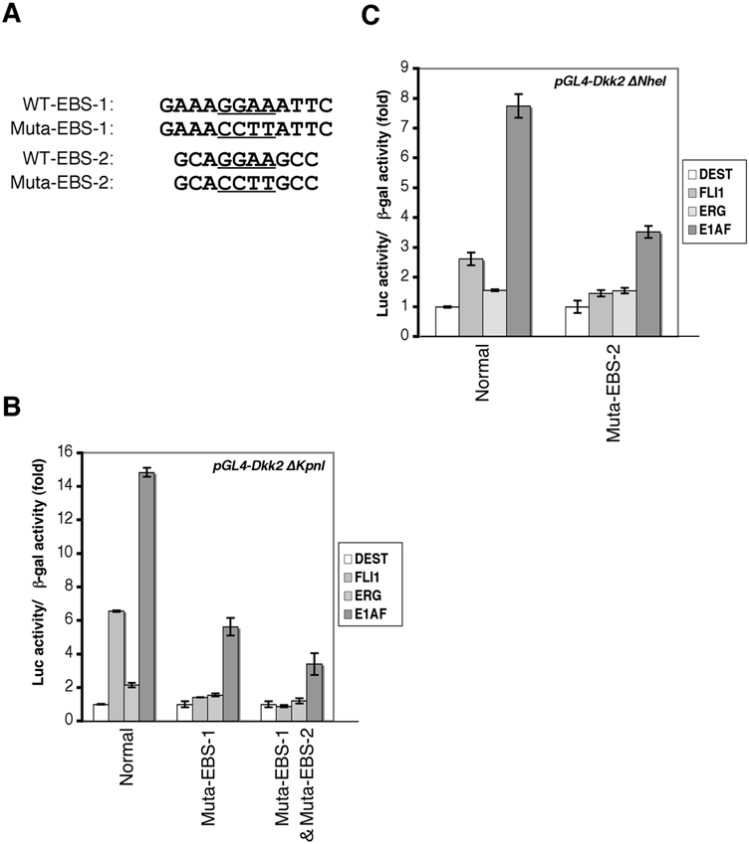
Mutational analysis of EBS in the DKK2 promoter. A, The substitutions in EBS-1 and EBS-2. The underlined sequences indicate the mutated sequences in EBS. B, C, The suppression of EWS/ETS-mediated activation of the DKK2 promoter by the mutations. HEK293 cells were co-transfected with mutated reporter plasmids and the expression vector. Luciferase activity was measured after 48 hours. Data are relative values with the SD for triplicate wells. Data are normalized to the value for the empty vector (pcDNA3-flagDEST) that is arbitrarily set to 1. The transfection efficiency was normalized relative to the β–galactosidase activity (See Materials and Methods).

To determine whether the EBSs are involved in the EWS/ETS-induced transactivation of the DKK2 promoter activity, effects of mutagenesis of EBS-1 and EBS-2 were tested. As shown in Fig. 3A and 3B, the mutation of EBS-1 suppressed the EWS/ETS-induced enhancement of the reporter activity of DKK2Δ*Kpn*I. When both EBS-1 and EBS-2 were simultaneously mutated, the suppressive effect against EWS/ETS was further strengthened (Fig. 3B). Likewise, the mutation of EBS-2 suppressed the EWS/ETS-induced activation of the reporter activity of DKK2 Δ*Nhe*I-Luc (Fig. 3A, C).

**Figure 4:**
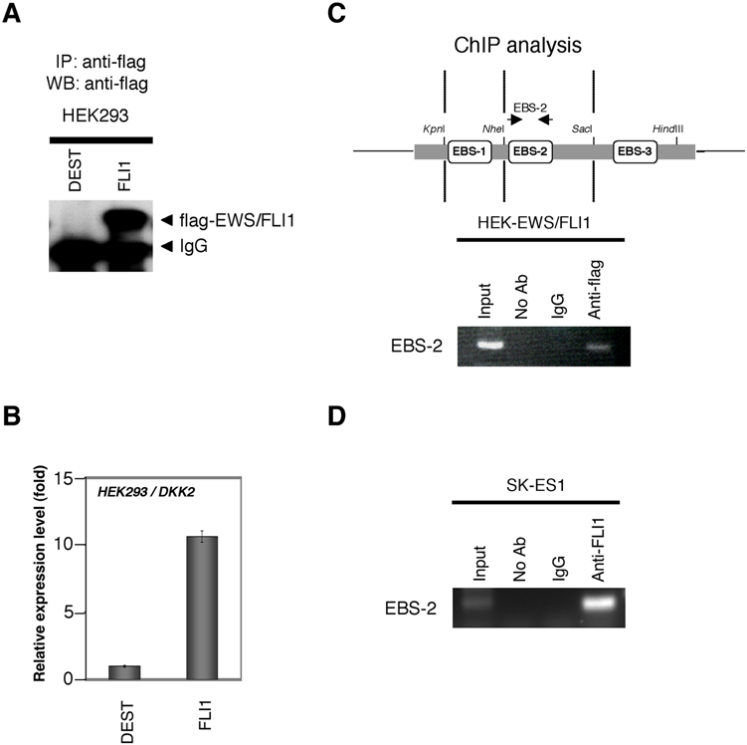
Direct binding of EWS/FLI1 to EBS of the DKK2 promoter. A, Immunoprecipitation (IP) and Western blot analysis of HEK293 cells stably transfected with pcDNA3-flagDEST (HEK-DEST) or pcDNA3-flagEWS/FLI1 (HEK-EWS/FLI1). Cells were lysed and Immunoprecipitated by anti-flag antibodies. The IP products were analyzed by Westen blot analysis. B, Real-time RT-PCR analysis using HEK293 transfectants for DKK2 expression. Data are normalized to the mRNA level in HEK-DEST cells which is arbitrarily set to 1. Signal intensity was normalized using that of a control housekeeping gene (human GAPDH gene). Data are relative values with the SD for triplicate wells. DEST: HEK-DEST cells; FLI1: HEK-EWS/FLI1. C, D, Binding of EWS/FLI1 to EBS of the DKK2 promoter *in vivo*. The ChIP analysis was done as described under Materials and Methods. For the ChIP analysis using HEK293 transfectants, soluble chromatin was immunoprecipitated with anti-flag antibodies or normal mouse immunoglobulin (IgG) and analyzed by PCR. For the ChIP analysis using SK-ES1 cells, soluble chromatin was immunoprecipitated with anti-FLI1 antibodies or normal rabbit immunoglobulin (IgG) and analyzed by PCR. The upper figure indicates a schematic representation of the site of the DKK2 promoter detected in the ChIP analysis. Arrows indicate the position of the specific primer used for ChIP.

We next evaluated the direct binding of EWS/ETS proteins to the EBSs in the DKK2 5′ upstream region. In this assay, HEK293 cells were stably transfected with pcDNA-flagDEST and pcDNA-flagEWS/FLI1, respectively. The overexpression of flag-tagged EWS/FLI1 protein was confirmed in the IP-western blot assay (Fig. 4A). DKK2 expression was significantly up-regulated in HEK-EWS/FLI1 transfectants, approximately 10 fold, compared with HEK-DEST cells (Fig. 4B). When the ChIP assay was performed using these cells as illustrated in Fig. 4C (upper figure), we found that EWS/FLI1 preferentially bound to EBS-2 in HEK-EWS/FLI1 transfectants (Fig. 4C). Similarly, we also demonstrated that EWS/FLI1 bound to EBS-2 in SK-ES1 cells (Fig. 4D).

We also examined the effects of EWS/ETS expression on DKK1 promoter activity. Although we also isolated the 5′ upstream region of the DKK1 gene (−2108/+80) and conducted reporter assays as for the DKK2 gene, no significant enhancement of DKK1 promoter activity mediated by EWS/ETS expression was observed (data not shown).

### Alteration of DKK1 and DKK2 expression by EWS/ETS in human MPCs

**Figure 5:**
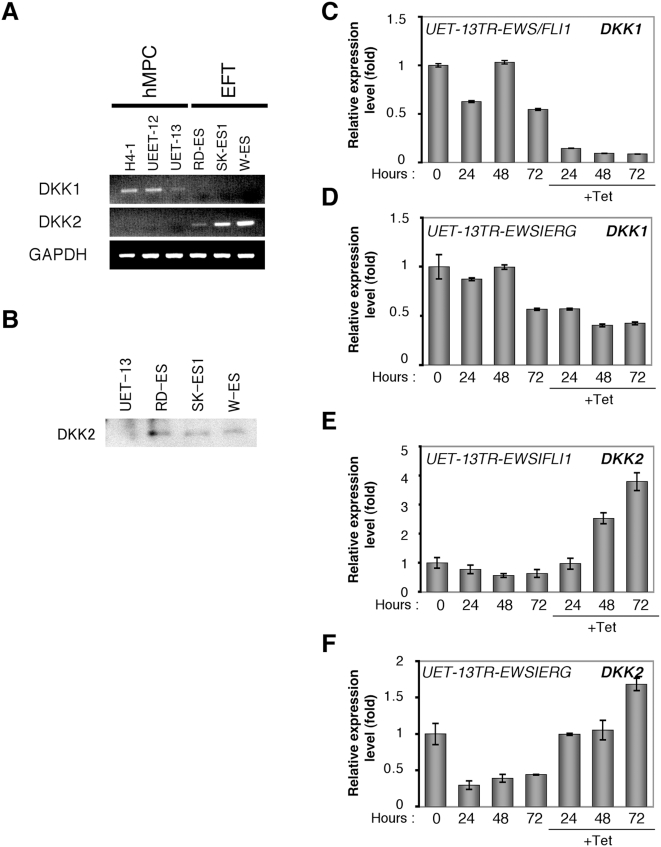
The change in expression pattern of the DKK family with EWS/ETS's induction in UET-13 cells. A, The expression pattern of DKKs in hMPCs and EFT cells. DKK1 and DKK2 mRNAs in the H4-1, UEET-12, UET-13 and EFT cell lines were detected by RT-PCR. As an internal control, a human GAPDH gene was used. B, The expression of DKK2 protein in hMPCs and EFT cells. For the detection of secreted DKK2, cells were cultured for 48 hours and then the culture medium was analyzed by Western blotting. C, D, E, F, Relative level of DKK1 (C, D) and DKK2 (E, F) in UET-13 transfectants in the absence or presence of tetracycline. UET-13 transfectants were treated with or without 3 µg/ml of tetracycline for the indicated periods. Real-time RT-PCR was performed to investigate the expression pattern of the DKK family. Signal intensity was normalized using that of a control housekeeping gene (human GAPDH gene). Data are relative values with the SD for triplicate wells and normalized to the mRNA level at 0 hour which is arbitrarily set to 1.

Although the origin of EFT is still unknown, some experimental results point to hMPCs. In hMPCs, DKK1 has a crucial role in proliferation and osteogenic differentiation [46], [47]. As shown in Fig. 5A, DKK1, but not DKK2, was expressed in primary hMPCs, H4-1 cells, UEET-12 cells and UET-13 cells. The expression of DKK2 protein was also confirmed in EFT whereas not in UET-13 cells (Fig. 5B). In EFT cell lines, by contrast, DKK2, but not DKK1, was expressed. We previously reported that the tetracycline-dependent expression of EWS/ETS confers EFT-like phenotypes in UET-13 cells [35]. Therefore, we tested the change of DKK gene expression using this model. As shown in Fig. 5C–F, both EWS/FLI1 and EWS/ERG expression resulted in a considerable increase in DKK2 gene expression. It is worth noting to describe that the expression level of the DKK1 gene was significantly decreased after EWS/ETS expression in UET-13 cells.

### Mutually exclusive expression of DKK1 and DKK2 in SK-ES1 cells

**Figure 6:**
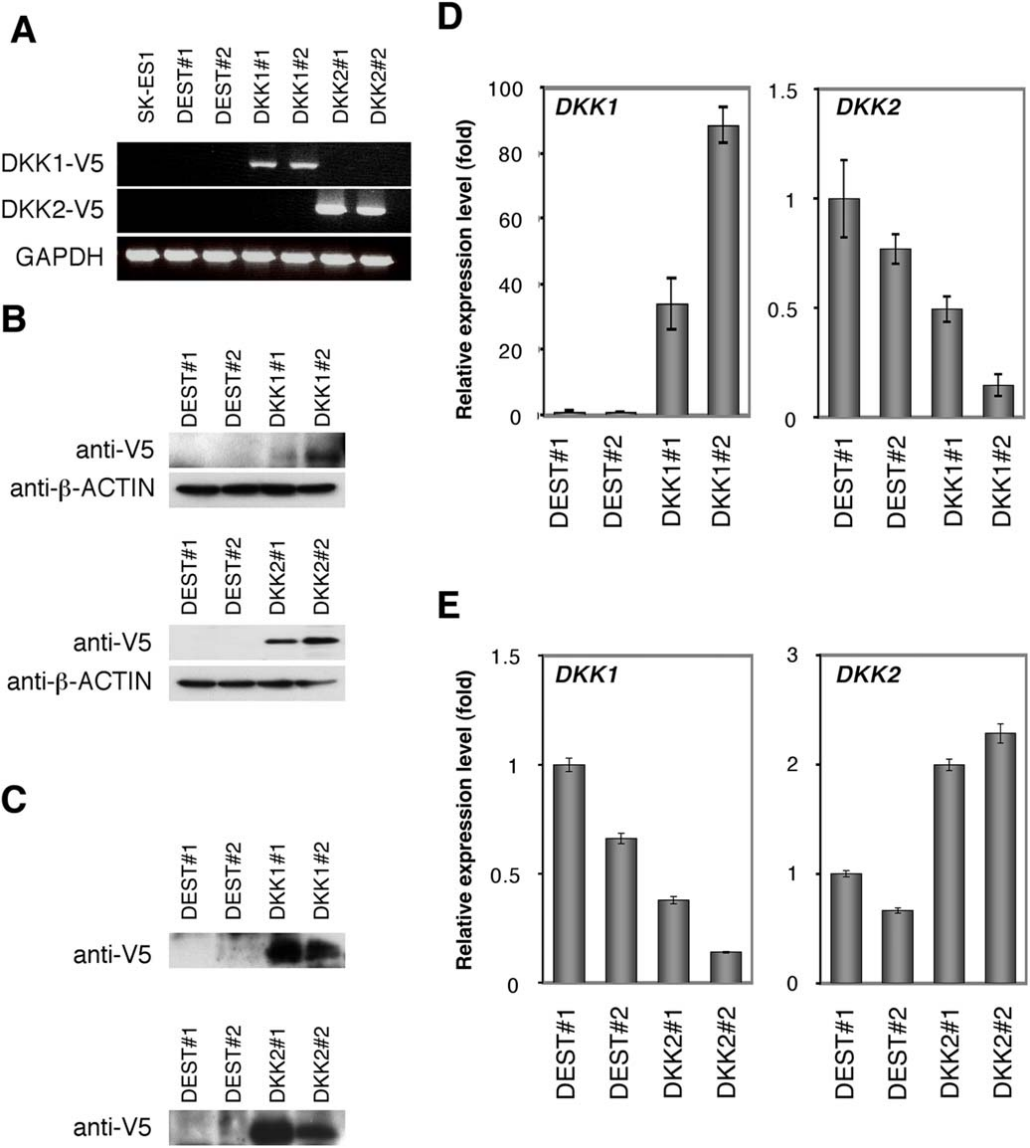
Mutually exclusive expression of DKK1 and DKK2 in SK-ES1 cells. A, B, Validation of DKK-V5 expression in SK-ES1 cells. The expression of DKK1-V5 and DKK2-V5 in SK-ES1 transfectants was observed by RT-PCR (A) and Western blot analysis (B). DEST#1, #2: SK-ES1-DEST#1, #2, DKK1#1, #2: SK-ES1-DKK1#1, #2, DKK2#1, #2: SK-ES1-DKK2#1, #2. As an internal control, human GAPDH (for RT-PCR) and β–Actin (for Western blot) were used, respectively. C, Secretion of DKKs-V5 into cell culture supernatants. Secreted DKKs-V5 proteins were absorbed by TALON Metal Affinity Resin (Clontech) and analyzed by Western blotting, D, E, Relative level of DKK1 and DKK2 in SK-ES1 transfectants in SK-ES1-DKK1 (D) and SK-ES1-DKK2 (E). Signal intensity was normalized using that of a control housekeeping gene (human GAPDH gene). Data are relative values with the SD for triplicate wells and normalized to the mRNA level at DEST#1 which is arbitrarily set to 1.

To further elucidate the biological roles of DKK genes in EFT cells, we introduced the DKK1 and DKK2 genes into the EFT cell line SK-ES1, in which the level of DKK2 expression is relatively low (Fig. 1A). Independent transfectants were confirmed to indeed express each gene by RT-PCR and Western blotting (Fig. 6A–C). Interestingly, when the DKK1 transfectants were examined by real-time PCR analysis, a reduction in DKK2 expression was found (Fig. 6D). In contrast, DKK2 overexpression induced a reduction in DKK1 expression in SK-ES1 cells (Fig. 6E). These results suggested that the expression of DKK1 and DKK2 related reciprocally in a mutually exclusive fashion in SK-ES1 cells.

### Expression of DKK1 inhibits the formation of subcutaneous tumors

**Figure 7:**
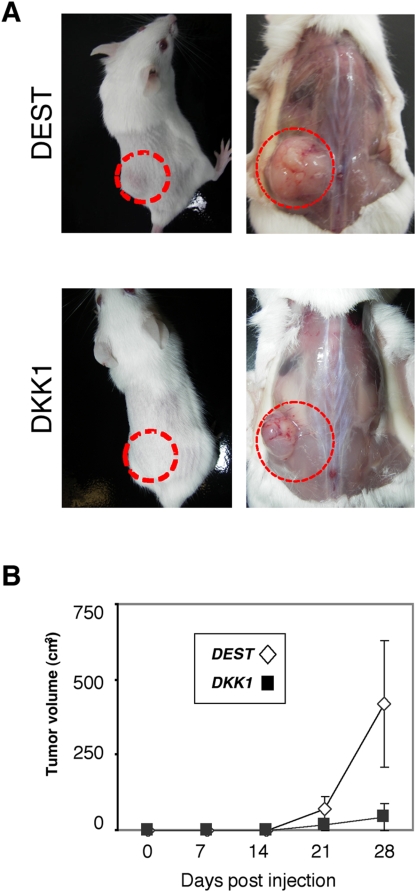
The effect of ectopic DKK1 expression on tumor cell growth *in vivo*. A, Examples of Immuno-deficient mice that have been injected with SK-ES1-DEST (DEST#1) and SK-ES1-DKK1 (DKK1#1) (left panels). The image was taken at 28 days after injection. Right panels indicate the xenografts of mice injected with DEST#1 and DKK1#1. Red circles indicate the positions of tumors. B, Tumor growth rates from mice injected with DEST#1 and DKK1#1. Diamond symbols indicate the tumor volume of mice injected with DEST#1; Box symbols indicate the tumor volume of mice injected with DKK1#1.

We next investigated the effect of DKK1 and DKK2 expression on tumorigenicity by subcutaneously implanting SK-ES1 transfectants into CB17/SCID mice. SK-ES1-DEST#1 and SK-ES1-DKK1#1 were implanted subcutaneously into CB17/SCID mice. The mice were sacrificed 28 days after injection. Four of the 10 mice injected with SK-ES1-DEST cells developed tumors (experiment #1 in Table. 1, Fig. 7A), in the case of SK-ES1-DKK1#1 cells, however, only one tumor mass was observed after similar preparation. In addition, the xenograft of DKK1-expressing SK-ES1 cells was significantly smaller than that of control SK-ES1-DEST cells (Fig. 7A, B). There were no morphological and pathological differences between xenografts formed by SK-ES1-DEST#1 and -DKK1#1 cells. Reproducible results were obtained by using another independent clone, SK-ES-DKK1#2 cells (experiment #2 in Table. 1). We also tested the tumorigenicity of SK-ES1-DKK2 cells in a similar manner. Although SK-ES1-DKK2#2 xenografts were significantly larger than control SK-ES1-DEST xenografts, the clone SK-ES1-DKK2#1 presented no significant difference from SK-ES1-DEST clones (data not shown).

**Table 1:** Formation of subcutaneous tumors in CB17/SCID mice by SK-ES1 cells.

Cells	Number tumors/ number injected	
	Experiments#1	Experiments#2
**DEST**	4/10	5/5
**DKK1**	1/10	2/5

## Discussion

In this study, we found that the expression of DKK2 was enhanced but that of DKK1 was suppressed in EFT cells. The suppression of DKK1 expression has been observed in several tumors [16], [48], [49], [50], and although elevated level of the DKK family occur in a number of tumor cells, most reports to date have been concerned with DKK1 and DKK3 [15]. In the case of DKK2, its suppression in tumors such as malignant melanoma [17] and gastrointestinal tumors [51] has been reported, but the elevated levels in cancer have not. Hence, the expression profile of the DKK family observed here is suggested to be specific to EFT.

We also demonstrated that the expression of DKK2 is regulated by the direct interaction of the chimeric protein EWS/ETS with EBS located in the promoter region of the gene. In contrast, although we also investigated the effect of EWS/ETS on DKK1 transcription, EWS/ETS exhibited no significant effect on DKK1 promoter activity as assessed by the reporter assay. However, the induction of EWS/ETS expression in human MPCs resulted in not only an increase in DKK2 gene expression but also a considerable decrease in DKK1 gene expression. Moreover, introduction of the DKK2 gene induced a reduction in DKK1 gene expression and vice versa in SK-ES1 cell. Taken together, our results indicate that EWS/ETS expression in EFT cells directly induces the up-regulation of DKK2 gene expression that consequently leads to a suppression of DKK1 expression, although the further analysis is required for understanding the underlying mechanism of EWS/ETS-mediated modulation of DKK family expression.

Although DKK1 is known as a Wnt antagonist, the function of DKK2 is still controversial. Some studies indicated that DKK2 also acts as an antagonist of Wnt signaling, whereas others showed that DKK2 behaves rather agonistic to Wnt signaling. In some tumor types, Wnt antagonists including DKK1 act as tumor suppressors [19], [20], [24], [52], [53]. As we demonstrated in this study, ectopic expression of DKK1 in EFT cells resulted in the inhibition of tumor growth in SCID mice, whereas DKK2 expression did not and even possibly accelerated tumor growth *in vivo*, demonstrating that DKK2 does not act as a counterpart of DKK1. Therefore, our findings support reports that presented DKK2 has a distinct function from DKK1. As we described above, the up-regulation of DKK2 consequently leads to a suppression of DKK1 and thus possibly contributes to EFT phenotype indirectly by interfering with the suppressive function of DKK1 on tumor growth. To further investigate the role of DKK2 in EFT formation, we have tested the effect of temporal DKK2 knockdown by transient transfection with DKK2 siRNA, whereas no significant influence on cell growth and cell death was observed as assessed by MTT assay and Annexin V assay, respectively (data not shown). The further experiments to elucidate the direct effect of DKK2 on EFT phenotype are now underway.

Interestingly, it was recently suggested that DKK1 also functions independently of the canonical Wnt/β–catenin signaling pathway. For example, several studies have indicated that DKK1-mediated tumor suppression is independent of canonical Wnt/β–catenin signaling [20]. It was also reported that Wnt3A-mediated activation of the canonical Wnt/β–catenin signaling pathway induces neuritic outgrowth in EFT cells [54], and DKK1 mimics Wnt activity and induces neuritogenesis in EFT cells. Therefore, it is possible that DKK2 also has a function independent of Wnt signaling that is distinct from that of DKK1.

As described above, DKK1 has a significant function in hMPCs and could stimulate cell proliferation while maintaining the undifferentiated phenotype [55]. Therefore, adequate DKK expression is essential for the maintenance of the MPC phenotype. Although the origins of EFT are still unclear, hMPCs are thought to be a candidate [56], [57] and we previously showed that inducible EWS/ETS expression in human MPCs confers EFT-like phenotypes [35]. Since, as we found in this study, EWS/ETS expression induces the down-regulation of DKK1 expression and up-regulation of DKK2 in UET-13 cells, an expression profile of the DKK family typical of EFT cells, it is reasonable to speculate that EWS/ETS-mediated change in the DKK family expression profile in hMPCs is involved in the development of EFT. Consistent with this, interestingly, a recent study revealed that the expression of DKK1 is up-regulated in EWS/FLI1-silenced EFT cells [58], in which feature of the mesenchymal progenitor including the capacity to differentiation into the osteogenic and adipogenic lineages are conferred. Considering the significance of DKK1 expression in hMPCs, EWS/ETS-mediated DKK1 suppression could impair the characteristics of hMPCs and contribute to their transformation into EFT.
